# The Book World of Medicine and Science

**Published:** 1894-02-03

**Authors:** 


### THE BOOK WORLD OF MEDICINE AND SCIENCE.

### THE ENDOWMENT OF PUBLIC PRACTITIONERS.—II.*

THE requirements for entering the medical department of the navy are the same as those for the army, but the number of officers is smaller, not more than 416 being allowed on active service. Successful candidates receive a course of instruction in naval hygiene at Haslar Hospital on first entering the service. On entry a surgeon receives pay at the rate of 11s. 6d. a day, or £209 17s. 6d. a year, rising after four years' service to £246 7s. 6d. a year, and after eight years to £282 17s. 6d. At the end of these eight years he is required to pass an examination qualifying him for the rank of staff-surgeon, although, save under exceptional circumstances of service or professional merit, he does not attain the position of a staff-surgeon for twelve years. The exceptional promotions are not permitted to number more than one a year. A staff-surgeon on promotion receives a guinea a day, or £383 5s. a year, and after four years' service £1 4s. a day, or £438 a year. Twenty years' service is requisite to qualify for the next rank, that of fleet-surgeon, though, as in the previous grade, exceptional promotions are permitted, not, however, more than one in two years. A fleet-surgeon on promotion receives £1 7s. a day, rising, after four years' service in the rank, to £1 10s., and, after eight years, to £1 13s., or, reckoning by the annual salary, £492 15s., £547 10s., and £602 5s. a year. Next above the fleet-surgeons stand the deputy-inspectors of hospitals, whose average number is only twelve. Their salary is two guineas a day, or £766 10s. a year. The highest rank is that of inspector-general, of whom only four are allowed. They receive £2 15s. a day—£1,003 15s. a year.

Besides this pay, certain allowances are granted. When serving afloat, surgeons receive an allowance of 1s. 6d. a-day in lieu of provisions, fuel, and lights, and there are graduated hospital allowances for medical officers at home and abroad. Thus a surgeon at home receives £39 a year allowance, and when abroad £108. Fleet-surgeons and staff-surgeons get £53 at home and £112 abroad; deputy-inspectors-general £67 at home and £112 abroad; and inspectors-general £85 at home and £130 abroad. The fleet-surgeon on board the flag-ship of a commander-in-chief on a foreign station receives a special allowance of 5s. a day, and the senior medical officer of a commodore's ship on a foreign station 2s. 6d. a day.

To obtain retired pay, a medical officer must have served for twenty years. If he retire before completing this term he receives only a gratuity. This gratuity after eight years' service is £1,000, after twelve years £1,500, and after sixteen years £2,000. If, however, an officer who has served less than twenty years retires on account of any disability contracted in and through the service, he may receive the half-pay of his rank, and throughout all the regulations regarding half-pay, windows' pensions, and children's allowances, special consideration is given to retirement or death through wounds or other disabilities received in the service. After twenty years' service a fleet-surgeon's retired pay is £365 a year, after twenty-four years £410 12s. 6d., after twenty-seven years £456, and after thirty years £547 10s. A deputy-inspector-general receives £638 15s. retired pay, and an inspector-general £730. Widows' pensions and children's allowance vary greatly, according not only to the circumstances, but to the time of retirement or death of the officer. The ordinary widow's pension rises from £50 for the widow of a surgeon, with £9 to £12 allowance for each child, to £120 for the widow of an inspector-general, and £16 to £20 to each child; but exceptional circumstances may, if the Lords of the Admiralty consent, procure larger pensions.

There are a number of medical appointments in the colonies, usually involving the care of a district, with hospital, asylum, poor-house, or other institution. The work is akin to that of a parish doctor at home, though over a wider district, and in most cases private practice is permitted. The rate of pay varies in different colonies. In Jamaica the salary averages £200 a year, while in British Guiana the lowest salary is £300, and rises to £900, with a travelling allowance of £100 to £150, according to the extent of the district. The salary of officers on the Gold Coast is larger than elsewhere, in consideration of the climate, and of the fact that private practice, though permitted, is very scanty. The colonial surgeon there receives £800, rising to £1,000, and the assistant-surgeons £350, rising to £400. In Jamaica and Ceylon vacancies are almost always filled up on the spot by the appointment of native-born candidates, and offices demanding specialist skill, as well as those head appointments which demand administrative as well as professional capacity, are usually filled up directly by the Colonial Office, without competitive examination.

There are several medical appointments in connection with the Civil Service. In every post office all employés whose salary is less than £150 a year have a right to be attended by the post office doctor. For this the doctor receives 8s. 6d. per annum for each individual under his care—even those who have not been ill. For itinerant members of the post office staff, such as telegraph linesmen, the medical officer is paid only for those who fall ill, but the pay is relatively higher. These appointments do not preclude private practice.

The pay of the medical officers of H.M. prisons varies with the duty. Where the doctor gives only part of his time to the work the salary is from £50 to £250 a year. Where all his time is devoted to it he receives from £250 to £500 a year and quarters. In Scotch prisons the maximum salary is £350 a year. Medical officers have also the right to a retiring pension not exceeding two-thirds of their former salary. Among the best paid Government medical appointments in Scotland are those of the Commissioners and Deputy-Commissioners in Lunacy. The pay of the former is £1,200, and of the latter £600 a year.

In Ireland there are four medical inspectors under the Local Government Board, with salaries of £500, rising to £700 a year. These inspectors receive also a personal allowance of a guinea a night, besides travelling expenses, when absent from head-quarters. The medical officers of health may also be regarded as public practitioners, although their appointments lie with County Councils and not the Crown. Their salaries vary with the extent of their districts and the onerousness of their duties. The remuneration is naturally larger where private practice is forbidden.

* See Faulkner's "Guide to the Public Medical Services." (London: H. K. Lewis, 136, Gower Street. Demy 8vo. Two Shillings.)

